# An X-ray free-electron laser with a highly configurable undulator and integrated chicanes for tailored pulse properties

**DOI:** 10.1038/s41467-023-40759-z

**Published:** 2023-08-21

**Authors:** Eduard Prat, Andre Al Haddad, Christopher Arrell, Sven Augustin, Marco Boll, Christoph Bostedt, Marco Calvi, Adrian L. Cavalieri, Paolo Craievich, Andreas Dax, Philipp Dijkstal, Eugenio Ferrari, Rolf Follath, Romain Ganter, Zheqiao Geng, Nicole Hiller, Martin Huppert, Rasmus Ischebeck, Pavle Juranić, Christoph Kittel, Gregor Knopp, Alexander Malyzhenkov, Fabio Marcellini, Stefan Neppl, Sven Reiche, Nicholas Sammut, Thomas Schietinger, Thomas Schmidt, Kirsten Schnorr, Alexandre Trisorio, Carlo Vicario, Didier Voulot, Guanglei Wang, Tobias Weilbach

**Affiliations:** 1 Paul Scherrer Institut, CH-5232 Villigen PSI, Switzerland; 2 Ecole Polytechnique Fédérale de Lausanne, CH-1015 Lausanne, Switzerland; 3 Institute of Applied Physics, University of Bern, CH-3012 Bern, Switzerland; 4 University of Malta, MSD2080 Msida, Malta; 5 Present Address: Deutsches Elektronen-Synchrotron, D-22607 Hamburg, Germany; 6 Present Address: CERN, CH-1211 Geneva 23, Switzerland

**Keywords:** Free-electron lasers, X-rays

## Abstract

X-ray free-electron lasers (FELs) are state-of-the-art scientific tools capable to study matter on the scale of atomic processes. Since the initial operation of X-ray FELs more than a decade ago, several facilities with upgraded performance have been put in operation. Here we present the first lasing results of Athos, the soft X-ray FEL beamline of SwissFEL at the Paul Scherrer Institute in Switzerland. Athos features an undulator layout based on short APPLE-X modules providing full polarisation control, interleaved with small magnetic chicanes. This versatile configuration allows for many operational modes, giving control over many FEL properties. We show, for example, a 35% reduction of the required undulator length to achieve FEL saturation with respect to standard undulator configurations. We also demonstrate the generation of more powerful pulses than the ones obtained in typical undulators. Athos represents a fundamental step forward in the design of FEL facilities, creating opportunities in FEL-based sciences.

## Abstract (web summary)

Since their initial operation, free-electron lasers are regularly upgraded in their performance and parameter control. Here the authors present the first lasing results of the soft X-ray free-electron laser beamline of the Paul Scherrer Institute, demonstrating different modes of operation and polarisation control of the tailored soft X-ray pulses.

## Introduction

X-ray free-electron lasers (FELs) are cutting-edge research tools employed in various scientific fields that allow the observation of matter on spatial and temporal scales of atomic processes^1–3^. The FEL radiation is produced by a high-brightness electron beam travelling through a periodic array of magnets called undulator. So far, there are eight X-ray FEL facilities in user operation worldwide: FLASH^4^ and the European XFEL^5^ at DESY in Germany, LCLS^6^ at SLAC in the USA, SACLA^7^ at RIKEN in Japan, FERMI at Elettra-Sincrotrone Trieste in Italy^8^, PAL-XFEL^9^ at PAL in South Korea, the SXFEL^10^ at SARI in China, and SwissFEL^11^ at PSI in Switzerland. In addition, several projects are in preparation like LCLS-II^12^ at SLAC in the USA and SHINE^13^ at SARI in China. Most of these facilities operate or will operate in the hard and soft X-ray regimes, except FLASH, FERMI, and the SXFEL, which are limited to soft X-rays. FLASH, the European XFEL, LCLS-II, and SHINE are designed for repetition rates at the MHz level, while the other facilities work at repetition rates at the level of 100 Hz.

Most of the X-ray FEL facilities are based on the self-amplified spontaneous emission (SASE) process^14,15^, a stochastic mechanism that starts from the electron beam shot noise and results in limited bandwidth and longitudinal coherence. Some of the facilities make use of the self-seeding mechanism to increase the longitudinal coherence and reduce the bandwidth of the SASE-FEL pulses (see e.g.,^16–18^). An alternative to self-seeding is external, laser-based seeding, with the advantages of a higher stability and synchronisation to an external source^19^. Moreover, since the properties of the input signal are mostly preserved by the FEL process, the final pulses can be tailored by the external signal and the electron bunch properties. FERMI and SXFEL are so far the only user facilities that employ laser-based seeding, currently limited to photon energies up to around 400 eV.

The FEL performance, in both SASE and seeding configurations, is defined by the saturation length and the saturation power of the FEL process. Higher electron beam brightness results in better FEL performance, i.e., a reduction of the saturation length and an increase of the saturation power. Moreover, helical undulators result in a stronger FEL coupling^2^ and therefore better FEL performance than standard planar undulators. In any case, the FEL saturation puts a fundamental limit to the maximum FEL output power, although techniques like undulator tapering can be employed to increase the FEL power beyond saturation^20^. The shortest FEL pulse duration is limited by the fraction of the electron beam that is suitable for lasing and ultimately by the FEL cooperation length, with typical values of a few hundred attoseconds for X-rays. Schemes based on superradiance can provide higher powers and shorter pulse durations than the standard FEL process^21–24^.

The polarisation of the X-ray FEL light depends on the type of undulator used to generate the radiation. There are mainly two types of undulators: planar, corresponding to linearly polarised light, and helical, providing circularly polarised radiation. Due to the simpler design, nearly all of the X-ray FEL facilities operate with planar undulators limited to linearly polarised light. The exception is FERMI, equipped with variable polarisation undulators^8^ to deliver variable polarisation light for photon energies up to around 300 eV. Finally, the standard operation of X-ray FEL facilities provides a single pulse. Two or more pulses, ideally with tunable delay and photon energy separation, are desired for certain applications such as X-ray pump X-ray probe experiments.

Here we present the first lasing results of the soft X-ray beamline of SwissFEL, Athos, at PSI in Villigen, Switzerland. The hard X-ray beamline of SwissFEL, Aramis, has been in regular user operation since 2019^11^. We report on the design of Athos and the demonstration of special modes providing precise control of the FEL properties, namely the saturation length, the polarisation, the peak power, the pulse duration, as well as multiple-pulse operation. Our results represent an important step towards a fully tailored X-ray FEL source, which will increase the opportunities for discoveries in multiple research disciplines.

## Results

### Design and layout

**Fig. 1:**
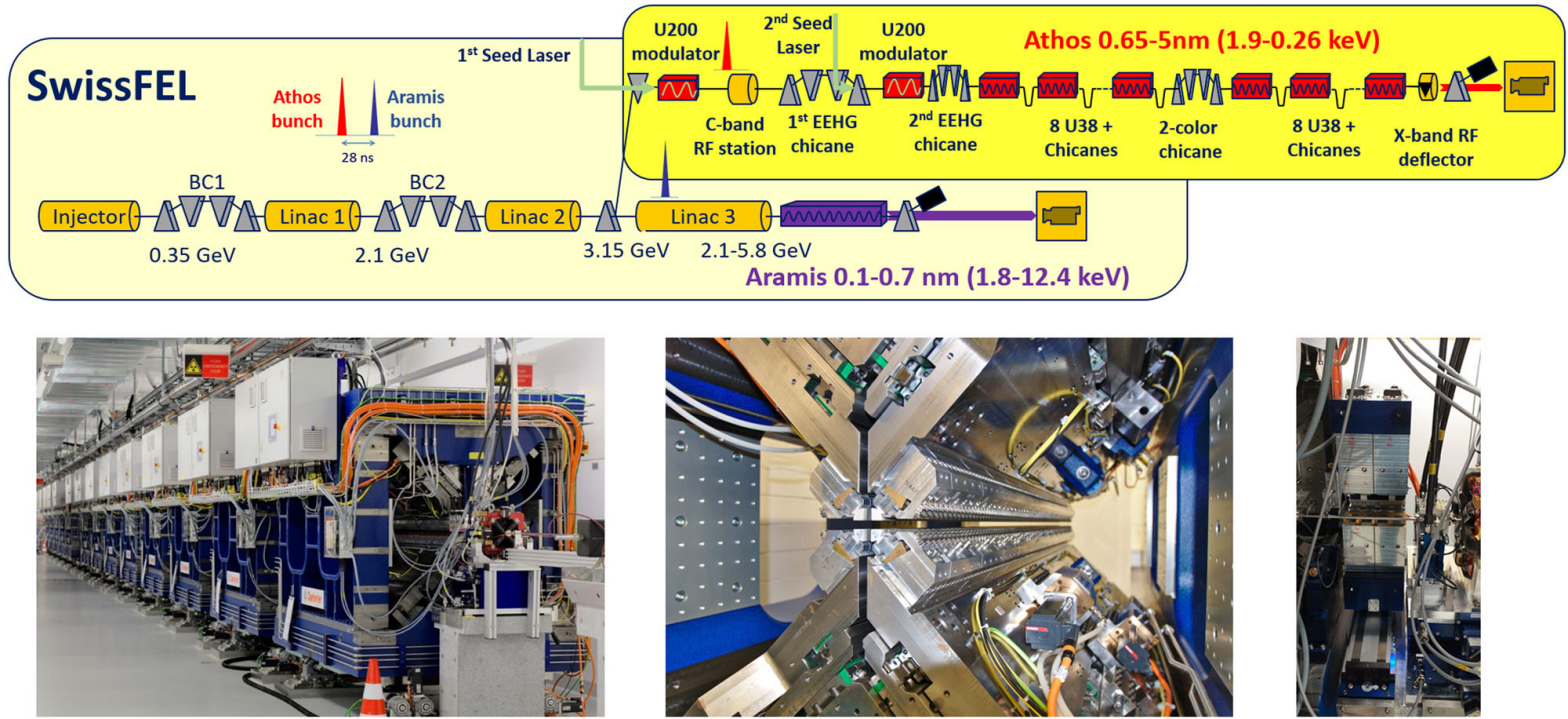
Athos sketch and images. Top: SwissFEL schematic (not to scale), adapted from Fig. 1 in Abela, R. et al. The SwissFEL soft x-ray free-electron laser beamline: Athos. J. Synchrotron Rad. 26, 1073–1084 (2019). Bottom: view of the undulator (left), picture of an undulator module (centre), and picture of an intra-undulator chicane installed in the tunnel (right).

Figure 1 shows an outline of the SwissFEL facility and images of the Athos undulator. The electron bunch for Athos has a typical charge of 200 pC, a duration between 15 and 30 fs (rms values) and normalised emittances of a few hundreds of nanometres as in Aramis. Athos offers two main innovations with respect to previous FEL designs. First, it employs an undulator type, called APPLE-X (U38)^25^, offering independent control of the undulator field strength, the photon polarisation and the transverse field gradient. There are 16 APPLE-X modules, each of them with a length of 2 m, a period of 38 mm, and a maximum deflection parameter *K* of 3.8. The transverse gradient allows the production of FEL pulses with ultra-large bandwidth^26^.

Second, there is a small magnetic chicane after every undulator module (except for the last one), capable of delaying the electron beam up to about 7 fs. These intra-undulator chicanes can be utilised to improve a number of FEL properties, namely to reduce the saturation length via the optical klystron (OK) effect^27^, to enhance the longitudinal coherence and reduce the bandwidth of the SASE-FEL pulses in the so-called high-brightness SASE scheme^28^, to obtain high-power short pulses via multi-stage amplification and superradiance^21–24^, and, in combination with an external optical laser, to generate trains of mode-locked attosecond pulses^29^.

Although Athos is based on the SASE process, it is also designed to employ optical lasers for external seeding. In particular, Athos aims to produce fully coherent pulses with the Echo-Enabled Harmonic Generation (EEHG) scheme^30^, so far demonstrated for photon energies of a few hundreds of eV^31^, up to the 1 keV level. The components for laser-based seeding are the optical laser system, two modulators (U200) with eight periods and a period length of 200 mm^32^, and two magnetic chicanes. At the time of writing, one optical laser amplifier was available, allowing the production of trains of attosecond pulses with the enhanced SASE (ESASE) mechanism^33^ or similar schemes (see Methods).

There is a large magnetic chicane in the middle of the undulator, employed to tune the delay (up to 500 fs) of two-colour pulses produced in a split-undulator configuration^34,35^, where each colour is produced in one half of the undulator. Other relevant components of Athos include a C-band radio-frequency (RF) station capable to tune the electron beam energy by ±250 MeV, i.e., between 2.9 and 3.4 GeV, and an X-band RF transverse deflector (TD) used for time-resolved diagnostics^36^.

The FEL fundamental wavelength *λ* depends on the electron beam energy and undulator parameters as^15^:

1 $$\lambda=\frac{{\lambda }_{u}}{2{\gamma }^{2}}\left(1+\frac{{K}^{2}}{2}\right)$$

The combination of the accessible beam energy range, the maximum *K* of 3.8, and a minimum *K* of 1 to have sufficient FEL coupling, corresponds to a wavelength range between 0.65 and 5 nm (or 1.9 to 0.26 keV), covering from the carbon K-edge to the silicon K-edge. The photon energy range of Athos complements that of Aramis well, which spans from 0.1 to 0.7 nm (1.8 to 12.4 keV).

Athos will provide FEL radiation to three endstations: Maloja, focused on atomic, molecular, non-linear, and chemical physics, in user operation since 2022; Furka, dedicated to quantum materials and currently performing commissioning experiments; and Diavolezza, which is currently in the design phase. Further details on the Athos design, operation modes, setup, and diagnostics are provided in Methods.

### First lasing and standard SASE

First lasing was achieved in December 2019 with only two undulator modules for a photon energy of 300 eV. Only after enabling the chicane between the two modules a small bright spot characteristic of FEL radiation (in comparison to the much larger and less intense spot due to spontaneous undulator radiation) could be seen. Consequently, the first lasing was also the demonstration of a special operation mode at Athos, namely the OK.

**Fig. 2:**
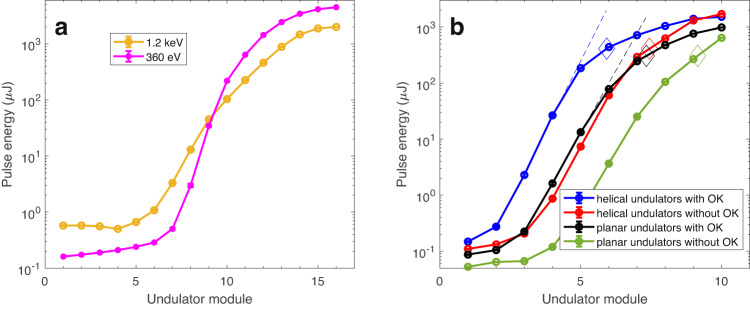
Measured FEL gain curves. The error bars represent one standard deviation of uncertainty. **a** Close to maximum achieved pulse energy for photon energies of 1.2 keV and 360 eV. **b** Comparison between helical and planar undulator configuration with and without OK for a photon energy of 545 eV. Circles indicate the measured values, solid lines connect the measurement points, dashed lines are exponential fits to the first undulator modules, diamonds correspond to saturation points.

After the consecutive installation of the remaining undulator modules in 2020 and the first half of 2021, with ongoing commissioning between installation periods, we have achieved pulse energies, as measured by the gas detector^37^, at the millijoule level for photon energies up to 1.4 keV. Up to now, maximum pulse energies for helical undulator configuration at Athos are 5 mJ for a photon energy of 500 eV, 4 mJ for 800 eV, and 3 mJ for 1 keV. Figure 2a shows FEL gain curve measurements for standard operation close to maximum pulse energies obtained at Athos for photon energies of 1.2 and 0.36 keV. For the 0.36 keV case, the first four undulator modules were open.

### Reduction of saturation length and increase of saturation power with helical undulators and optical klystron

More compact and higher-power FELs render these facilities more efficient, affordable, and accessible to the scientific community. For a given undulator length, the reduction of the saturation length means more space for tapering to increase the final pulse energy and opens up the possibility to reach higher photon energies, which require a longer undulator section. These effects are especially important for those operation modes requiring more undulator modules, such as the production of two-colour pulses in the split-undulator configuration. In fact, for Athos, the helical configuration and the OK are necessary for this mode when operating at high photon energies^35^.

Besides the single-stage OK, required for the first lasing, we have shown the impact of the OK distributed along the undulator to reduce the overall FEL saturation length^27^. Moreover, we have demonstrated that the saturation length can be further decreased with undulators in helical configuration instead of planar. We also show that helical undulators result in a higher FEL power. The OK was previously demonstrated at FERMI with a single stage in the EUV regime^38^ and with two and three stages for photon energies reaching into the soft X-ray regime^39^. An improvement of helical undulators in comparison to planar configurations was also shown at FERMI for EUV photon energies^40^. Now in Athos we show that the two effects can be combined effectively all along the undulator to achieve an enhanced improvement of the FEL performance in the X-ray regime.

Figure 2b shows the FEL gain curves measured for 10 undulator modules for a photon energy of 545 eV for four different configurations: helical undulators with and without OK, and planar undulators with and without OK. The setup of the chicanes was done stepwise following the procedure described in ref. ^27^. The curves indicate a clear benefit from applying the OK and from using helical undulators. The FEL process reaches saturation significantly earlier with helical undulator configuration and when the OK is applied. In addition, the final FEL pulse energy is higher for helical undulator configurations than for planar configurations (for both cases, with and without OK).

We define the FEL saturation point for the OK cases as follows: we fit the gain curve at the undulator modules with the highest gain with an exponential function and extrapolate it to the next modules. We then define the saturation point to be the location at which the pulse energy of the extended fit is five times larger than the measured pulse energy interpolated at that location. For the configurations without OK, we assume that the saturation pulse energy is the same as for the OK cases. The fits are shown as dashed lines in the plot, and the saturation points as diamonds. With this definition, the saturation pulse energies are 412 μJ for the helical undulator configuration, and 306 μJ for the planar one. The saturation lengths are 16.6 m for helical with OK, 20.9 m for helical without OK, 20.5 m for planar with OK, and 25.6 m for planar without OK. In comparison to the standard configuration based on planar undulators without OK, the saturation length is reduced by about 35% when using helical undulators and OK. Each effect, undulator configuration and OK, accounts for about half of the improvement. The saturation power is 35% higher for helical configuration than for planar. We repeated the same measurements for photon energies of 960 and 350 eV, obtaining similar results, in particular for the reduction in saturation length.

### Variable polarisation

Polarisation control opens a wide field of scientific applications ranging from linear and circular dichroism measurements for studying the properties of magnetic materials to the investigation of chiral molecules. The use of soft X-rays for dichroism experiments is particularly attractive, since a variety of highly relevant absorption edges, such as the metal L- as well as the carbon, nitrogen and oxygen K-edges can be addressed state-specifically.

At LCLS, a certain degree of polarisation control has previously been achieved with a variable-polarisation undulator module placed at the end of the undulator, combining reverse taper and beam-diverting techniques^41^. Starting from linear polarisation, the authors produced radiation with a few hundreds of microjoules and a 98% degree of circular polarisation. The drawbacks of this method are that it requires an FEL collimator and that the FEL pulse energy is significantly reduced. A better approach relies on all undulator modules having variable-polarisation capability. This option, besides not requiring any collimator and delivering higher pulse energies, gives us the capacity to produce tunable two-colour X-ray FEL pulses with different polarisations (using the split-undulator configuration). So far, this has only been accomplished at FERMI, which is capable of providing variable polarisation using APPLE-2-type undulators for photon energies up to 300 eV^8^. Athos is equipped with in-house designed APPLE-X undulator modules providing not only variable polarisation, but also independent control of the transverse field gradient. Moreover the polarisation control extends to photon energies up to the keV level.

The capacity of the Athos undulator to deliver FEL light with variable polarisation has been demonstrated experimentally. Here we report on the generation and measurement of linear horizontal, linear vertical, and circular polarisations for a photon energy of 700 eV. In our undulators, the zero shift position of its four independent magnet arrays is designed and calibrated as a planar configuration, generating a linear horizontal polarisation (defined as 0°)^42^. Starting from there, various polarisations can be produced by shifting the four magnet arrays against each other by two distinct movement patterns. To create linear inclined polarisations with an arbitrary angle (between −90° and +90°), we shift two opposite magnet arrays against each other (anti-parallel movement), while the remaining two arrays stay at their respective zero positions. In contrast, for elliptical and helical polarisations two opposite magnet arrays are shifted together (parallel movement) against the other two magnet arrays. We note that at the extremes of the parallel movement for the elliptical polarisations we naturally also generate linear horizontal and linear vertical polarisations. Up to now we exclusively employ the parallel movement in daily operation, since it covers everything but arbitrary linear polarisation angles.

**Fig. 3:**
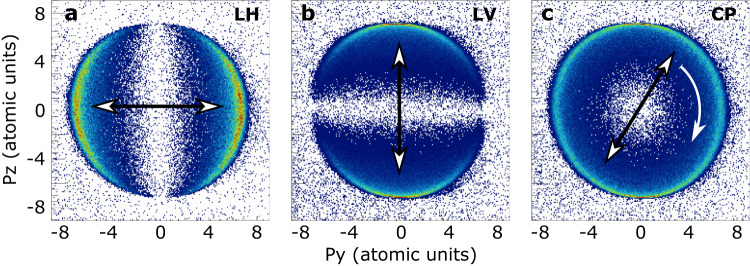
Measurement of variable polarisation for soft X-rays at 700 eV. The plots show the recoil ion momentum distribution of He^+^ ions projected onto the plane of the X-ray propagation direction with (**a**) linear horizontal (LH), (**b**) linear vertical (LV), and (**c**) circular polarised (CP) radiation. The arrows indicate the polarisation direction. The radius of the distribution corresponds to the absolute momentum of 7 a.u. (atomic units) imparted by the photoelectron onto the recoil ion.

To characterise the polarisation of the soft X-ray radiation we exploit the well understood single photoionization characteristics of He atoms using recoil ion momentum spectroscopy^43^. Following ionisation, the emitted photoelectron carries an energy equal to the incoming photon energy minus its binding energy. Like in any ionisation process, the observed momenta of the reaction fragments (here: recoil ion and photoelectron) are connected by momentum and energy conservation. Thus, the momentum of the ejected electron *p*_*e*_ is transferred to the remaining ion as recoil momentum *p*_*r*_ (*p*_*r*_ = − *p*_*e*_). Therefore, the angular emission pattern of the photoelectron distribution is imprinted in the recoil ion distribution. For perfect linearly polarised light and under the dipole approximation, the photoemission pattern of He is characterised by a *β* parameter of 2^44^, which corresponds to a dipole distribution along the polarisation axis. In the measured recoil ion momentum, this manifests as a double-lobed distribution for linear polarisation (Fig. 3a, b) and a sphere for circular polarisation (Fig. 3c) due to the rotating polarisation vector. The shown ion momentum distributions are projected onto the plane perpendicular to the X-ray propagation direction. Due to a higher momentum resolution in the *z*-direction, given by the measurement geometry, the distribution is narrower in that direction and wider in the *y*-direction.

### Single, two-colour and high-power short FEL pulses

Single- and two-colour short pulses are beneficial for multiple applications. In particular, widely tunable two-colour pulses, where the time and photon-energy separation between the two pulses can be varied over a large range, offer opportunities to study ultrafast X-ray-induced energy transfer and relaxation processes in physics, chemistry, and biology. Moreover, certain applications such as nonlinear optics and imaging experiments require not only short FEL pulses but also high power. Such high-power short FEL pulses are very attractive for these applications.

The standard FEL pulse duration giving maximum pulse energy is between 15 and 30 fs (rms values). Shorter pulses with tunable duration down to a few fs are regularly obtained by transversely tilting (or, more correctly, streaking) the electron beam^45^. In Athos, the beam can be tilted in the horizontal plane by inducing dispersion with a quadrupole magnet in the transfer line between the main SwissFEL accelerator and the Athos branch. There, the electron beam has an energy chirp, which, in combination with dispersion, results in a beam tilt. Varying the quadrupole magnet field allows us to change the amplitude of the tilt and thus the photon pulse duration – the larger the tilt amplitude, the shorter the photon pulse (and the lower the pulse energy).

In addition to this straightforward generation of short pulses the tilted electron beam is also used for two more advanced modes (see more details in Methods). In the first advanced mode, we tilt the beam to produce widely tunable two-colour short FEL pulses in a split-undulator configuration with the fresh-slice technique: the tail of the bunch is well aligned and produces one colour in the first undulator half, while the head of the bunch is well aligned and generates the second colour in the second half. This was reported earlier by the LCLS team^46^. We have advanced the scheme by increasing its tunability and by making it more compact (with the OK). Moreover, as mentioned earlier, we can also generate tunable two-colour X-ray FEL pulses with different polarisations. Thanks to our large *K* tuning range, we have demonstrated the production of two-colour FEL pulses with large tunability^35^, showing an energy ratio between the two pulses of about a factor of three (0.35 and 1 keV). The time separation between the two colours can be tuned with the large chicane up to about 500 fs. In the second advanced mode using a beam tilt, high-power short pulses are generated with the multi-stage fresh-slice amplification scheme^23^. This method was first demonstrated at LCLS with three amplification stages^47^, limited by the number of chicanes (two) present in the undulator. At Athos we have advanced the method by demonstrating four-stage amplification, with the potential to exploit up to six stages with the present number of undulator modules^48^.

**Fig. 4:**
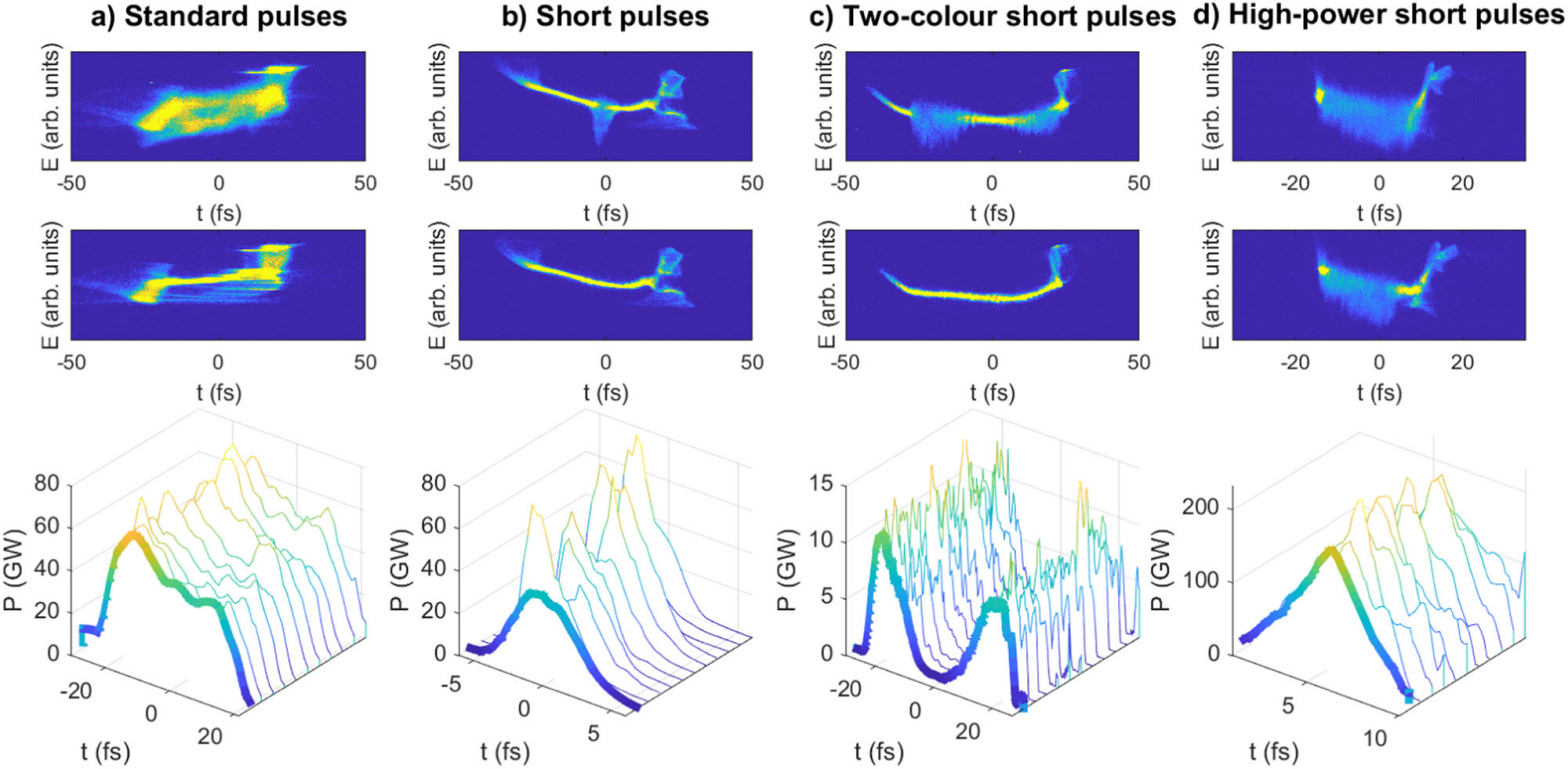
FEL power profile reconstruction for four different configurations. **a** standard pulses, where the whole bunch is lasing, (**b**) short pulses with standard power, (**c**) two-colour short pulses, and (**d**) high-power short pulses. Top plots: single-shot images for lasing-on conditions. Middle plots: single-shot images for lasing-off conditions. In the images, *E* indicates the energy of the electrons in arbitrary units, and *t* the time in femtoseconds (with *t* > 0 corresponding to the head of the bunch and *t* < 0 to the tail). Bottom plots: FEL power profiles reconstructed by comparing lasing-on and -off conditions (the bold profiles show average profiles, the rest correspond to single-shot power profiles). See text and Methods for more details.

Figure 4 shows the FEL power profile reconstruction for four different configurations. The first represents the standard operation mode, for which practically the whole bunch is lasing at a photon energy of 900 eV. The second case is for the short-pulse mode using a tilted beam for a photon energy of 900 eV, starting from the previous configuration. The third configuration corresponds to two-colour operation using the fresh-slice technique, for photon energies of 500 and 760 eV. And the fourth example illustrates the high-power short-pulse mode achieved with the multi-stage amplification technique for a photon energy of 650 eV. For the results reported here with this last configuration, a short pulse was amplified in four stages: the first stage consists of the first seven modules, while each of the following stages comprises three undulator modules. The pulse energies for the different cases were 2 mJ for the standard operation mode, 0.2 mJ for the short pulse mode, 0.12 mJ per each pulse in the two-colour mode, and 0.82 mJ for the multi-stage amplification configuration.

The FEL power profile is obtained by comparing the longitudinal phase space (LPS) of the electron beam, measured on a screen^49^, between lasing-on and lasing-off conditions. The upper plots show single-shot LPS images for lasing-on conditions, the middle plots display single-shot LPS images for lasing-off conditions, and the lower plots show the average FEL power profile (in bold) and 10 single-shot reconstructions. In all cases, the time axis is obtained with the X-band TD. See Methods for more details on the FEL power profile reconstruction.

For the multi-stage amplification method, the lasing-on conditions correspond to the end of the undulator, while the lasing-off conditions are after the third amplification stage, so the reconstructed FEL power profile is the one at the end of the beamline. LPS images at the other amplification stages and the FEL gain curve, not shown here, indicate a successive amplification of the FEL pulse over the four stages.

The reconstructed peak powers and rms pulse durations (obtained from Gaussian fits) are 66 ± 6 GW and 15.6 ± 0.9 fs for the full-bunch lasing configuration, 60 ± 14 GW and 1.4 ± 0.1 fs for the short pulse mode, 11 ± 2 GW and 5.1 ± 0.7 fs for colour one and 13 ± 1 GW and 4.5 ± 0.4 fs for colour two in the two-colour configuration, and 190 ± 25 GW and 2.0 ± 0.3 fs for the high-power short-pulse mode. As expected, the multi-stage amplification approach is useful to produce more powerful FEL pulses with respect to the other, more standard configurations. The peak power for the multi-stage amplification approach is about a factor of three higher than in the standard configurations, demonstrating the capacity of this approach to go beyond the FEL saturation power.

### Laser-based generation of attosecond pulse trains

Trains of X-ray attosecond pulses, preferentially if phase-locked, may pave the way for scientific opportunities in X-ray spectroscopy techniques, such as resonant inelastic X-ray scattering, and time-domain interferometry applications, such as RABBIT^50^.

We have performed a proof-of-principle experiment showing the generation of trains of short FEL pulses for a photon energy of 1 keV. We applied two methods (see Methods for more details): (1) laser-based energy modulation and compression (ESASE), for an external laser with a wavelength of 790 nm, and (2) energy modulation only, for laser wavelengths of 790 and 390 nm. In ref. ^51^ ESASE was already shown for a periodicity of 2 μm. We note that trains of phase-locked attosecond pulses have been demonstrated using a different technique in the EUV regime^52^.

In the ESASE configuration, the electron beam carries a density modulation with expected peak currents on the order of 10 kA. This generates strong space-charge forces, resulting in a distinct energy chirp along the current spikes. We compensate for it with a strong positive undulator taper to preserve the resonance condition while the radiation spikes slip along the energy chirp. In the experiment, where the second half of the undulator was used, we achieved the best performance with an FEL pulse energy of 150 μJ for a taper amplitude of 5%. Both laser modulation and positive taper were required to achieve significant lasing, which allows us to exclude any parasitic SASE process obscuring the FEL output signal.

A train of attosecond pulses gives rise to side-band patterns in the spectrum with a band separation corresponding to the modulation wavelength of the external laser. The single-shot spectrum is still dominated by the random spike pattern of the underlying SASE process. Moreover, the width of the SASE spectrum may be larger than the band separation. To overcome these difficulties, we gathered hundreds of spectra and calculated the second order spectral correlation function

2 $${g}_{2}(\omega,\Delta \omega )=\frac{\langle I(\omega )\cdot I(\omega+\Delta \omega )\rangle }{\langle I(\omega )\rangle \langle I(\omega+\Delta \omega )\rangle },$$

where *I*(*ω*) is the spectral intensity at a given frequency *ω*. We then average over all frequencies *ω*, where the average spectral intensity 〈*I*(*ω*)〉 lies above a fraction of the peak spectral intensity. In our case we apply thresholds of 90%, 70%, and 50%.

**Fig. 5:**
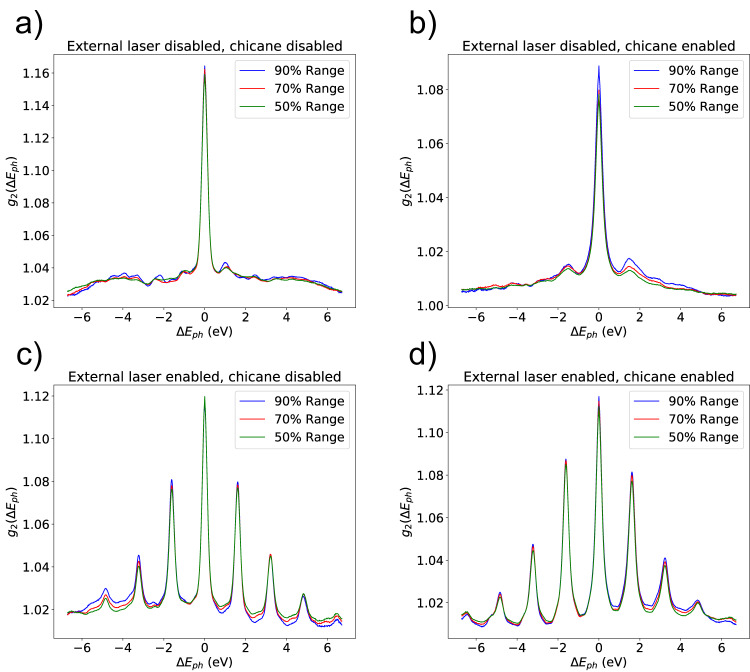
Measurements of spectral correlation functions for different conditions. **a** without external optical laser and with intra-undulator chicanes disabled, (**b**) without external optical laser and with matching mode-locked lasing condition, (**c**) with external optical laser and with intra-undulator chicanes disabled, (**d**) with external optical laser and with matching mode-locked lasing condition. See text for more details.

The spectral correlation functions for the cases of the energy modulation configuration and an external optical laser wavelength of 790 nm are shown in Fig. 5. In this case, we also set up the machine for mode-locked lasing, where the delay of the intra-undulator chicanes and the FEL slippage within one undulator period matches the modulation wavelength of 790 nm. In particular, we optimised the chicane delay equivalent to a value of 760 nm. Figure 5 shows four different cases depending on whether the external optical laser and the chicanes are enabled or disabled. When the optical laser is on (bottom plots), the spectral correlation functions show, as expected, the side-band signature of a train of short pulses with frequency peaks with the periodicity of the seed laser period (790 nm or 1.57 eV in terms of photon energy). We observe up to four sidebands on each side of the correlation measurement, indicating that the duration of each individual pulse should be shorter than the seed laser period over four (0.67 fs). Our simulations indicate that the duration of each individual pulse is between 0.4 and 0.5 fs (FWHM values).

For the two cases where the external laser is enabled, we see no significant difference when the chicanes are set either to a delay for mode-locked lasing or to no delay at all for ESASE. This is expected since the spectral correlation mostly indicates the smallest spectral coherent feature in any FEL signal, which is the FEL pulse duration, longer than the coherence length of the mode-locked lasing. Phase sensitive measurements, not available at the time of the experiment, will be required to prove mode-locked lasing. Nevertheless, for the case where the optical laser is disabled but the chicanes are enabled (top-right plot), we observe a weak sideband in the spectra at around 1.57 eV, corresponding to the periodicity of 790 nm. The observed sidebands indicate the ability of the FEL to duplicate SASE spikes, an important step towards mode-locked lasing.

## Discussion

We have presented the first lasing results of Athos, the soft X-ray beamline of SwissFEL. Athos is a facility with a highly flexible undulator and intra-undulator magnetic chicanes. We have shown control of several FEL properties such as polarisation, saturation length, pulse duration and power. Athos is the only current facility with integrated chicanes all along the undulator that can exploit some schemes such as the OK or the multi-stage amplification method to the full extent. Athos will also offer external seeding for photon energies that can potentially reach 1 keV and higher. All in all, Athos represents a fundamental step towards an ideal X-ray FEL source approaching standard table-top optical laser flexibility, where researchers can fully tailor the radiation properties to the specific needs of the experiment, opening the door to scientific opportunities and discoveries.

Besides further improving the already demonstrated operation modes, future efforts will focus on demonstrating further modes to tailor properties such as the longitudinal coherence and bandwidth. In particular, we plan to demonstrate the high-brightness SASE scheme, the EEHG mechanism, and the production of ultra-large bandwidth FEL pulses using the transverse-gradient option of our undulators^26^.

## Methods

### Design and operation modes

The Athos undulator consists of 16 APPLE-X undulator modules^25,42^. The APPLE-X was designed to provide independent and straightforward control of the undulator field strength, polarisation and transverse gradient. This is achieved with four magnet arrays individually adjustable in the radial direction, at an angle of 45° relative to the vertical plane, as well as in the axial direction. The photon energy and the polarisation of the emitted radiation can then be tuned independently with eight motors. The result is a fully symmetric design with equal vertical gaps and horizontal slits for all undulator field values. Furthermore, it is possible to operate asymmetrically, e.g., with left and right arrays at different gaps to generate a transverse magnetic field gradient. The length of each undulator module was chosen to be 2 m for best performance for various proposed operation modes^48^, significantly shorter than in standard FEL facilities. The undulator modules have a period of 38 mm and a maximum deflection parameter *K* of 3.8.

Variable polarisation is beneficial for a number of applications such as dichroism experiments. Without any specific request on the polarisation, it is preferable to have the undulators operate in helical configuration (corresponding to circular polarisation), since it results in stronger FEL coupling with respect to planar undulators (corresponding to linear polarisation). Finally, a transverse gradient is advantageous for two reasons. First, it can be employed to produce ultra-large bandwidth radiation above the 10% level^26^, which can be beneficial for some applications like spectroscopy and crystallography. Second, a transverse gradient with a variable gap can be used to generate a continuous field taper within each module by transversely tilting the module. In comparison to the standard stepwise tapering, for which the field is constant within an undulator module, a continuous taper allows the extraction of more FEL pulse energy and it increases the efficiency of certain laser-based techniques requiring tapered undulator fields^53^.

The space between the undulator modules measures 0.8 m, and contains a compact magnetic chicane, a quadrupole magnet to focus the electron beam, dipole corrector magnets to steer the electron beam trajectory, electron beam diagnostics, and vacuum components. The intra-undulator chicanes consist of four permanent dipole magnets occupying a total space of 0.2 m. The four dipoles act in the horizontal direction. The chicanes, when active, induce longitudinal dispersion and a delay to the electron beam, with the longitudinal dispersion corresponding to approximately twice the equivalent delay (path length difference). Each half of the chicane is motorised independently, thus giving the option to produce a transverse (horizontal) offset when the two halves have different gaps. The chicanes can provide a maximum delay of about 7 fs and a horizontal offset of ±400 μm. Moreover, they have sufficient precision to act as phase matchers as well. To support proper phase matching in transverse gradient mode, the chicanes can be shifted transversally to be operated in the roll-off region of the field, thus also providing some gradient. The chicanes offer at least four benefits. First, the longitudinal dispersion is useful to accelerate the FEL process and thus to reduce the saturation length via the distributed OK (see^27^ and references herein). Second, the delay can be employed to enhance the longitudinal coherence and reduce the bandwidth via the high-brightness SASE process^28^. Third, in combination with an external optical laser, applying a delay equivalent to the laser wavelength gives the option of delivering trains of mode-locked attosecond pulses^29^. And fourth, the delays give the possibility to apply the multi-stage amplification mechanism after each module to reach shorter and more powerful pulses following the superradiance approach^21–24^.

In the multi-stage amplification approach, a short pulse is consecutively amplified by different fresh parts of the electron beam in different stages. This scheme is based on tailoring electron beam properties such as the peak current^21,24^, the emittance^22^, or the trajectory with a transverse tilt^23^. Tilting the beam is the most efficient approach, in the sense that more electrons contribute to the lasing. In this option, the tail of the bunch is well aligned and produces a short FEL pulse at the first undulator stage, while the rest of the bunch travels off-axis and does not produce FEL radiation. The short pulse is then consecutively amplified by the other parts of the bunch in the following stages. For this to work, the fresh electrons must be brought to overlap with the previously generated FEL pulse. The longitudinal overlap is achieved by delaying the electron beam with the intra-undulator chicanes. For the transverse overlap, the fresh electrons are aligned using a dipole corrector and a transverse offset, the latter accomplished again by the intra-undulator chicanes.

In addition to the small chicanes, a two-metre long magnetic chicane, capable of delaying the electron beam up to about 500 fs, separates the undulator into two halves. This large chicane is useful to tune the time delay between the two colours produced in the split-undulator configuration. In this scheme, each colour is generated in one half of the undulator. The photon energy separation between the two pulses is tuned by adjusting the magnetic strength of each undulator section, while the time separation is tuned with the magnetic chicane installed between the two undulator sections. This mode has two variations: standard and fresh-slice. In the standard configuration the whole bunch lases in both undulator sections. By contrast, in the fresh-slice mode only the tail of the bunch is made to lase in the first undulator stage, whereas only the head of the bunch lases in the second undulator section. The fresh-slice option is preferable for several reasons: the time between the two pulses can be set to zero (both pulses arriving at the same time) or even negative values (the first colour arriving after the second), the pulses are shorter and the output of both pulses is more stable and mostly uncorrelated.

Components for laser-based seeding are installed in the straight section in front of the undulator. Athos aims at exploiting external seeding with the EEHG scheme^30^ for photon energies up to the 1 keV level. The required components are the optical laser system, two modulators with 8 effective periods of 200 mm period length^32^, and two magnetic chicanes. The seed laser system is a terawatt-class, femtosecond laser system based on titanium-sapphire technology. It consists of two parallel chirped pulse amplifiers seeded with a common laser oscillator. Each of the two laser amplifiers produces up to 10 mJ, 25 fs FWHM (Fourier-transform limited) pulses. The laser central wavelength can be tuned by ±2% around the central wavelengths of 790 and 390 nm. This spectral tunability is achieved using an acousto-optic programmable gain-control filter inside the regenerative amplifier together with the use of an acousto-optic programmable dispersive filter before the amplifier. Thanks to the latter, the pulse duration can be freely changed without affecting the pulse arrival time at the modulators.

At the time of writing, the first laser amplifier, the first magnetic chicane and the two modulators have been installed. This configuration can be employed to produce trains of attosecond pulses in two different ways. First, in the so-called ESASE scheme^33^, the optical laser induces, in the field of the modulator, an energy modulation to the electron beam with the period of the laser wavelength. After the chicane, the energy modulation is converted into a density modulation. The electron beam with its multiple, regularly spaced current peaks produces a train of short pulses in the undulator. An alternative to ESASE consists in combining the energy modulation with a strong taper of the undulator field, as proposed in ref. ^53^. Regardless of the method, the short pulses of the train can be phase locked if the intra-undulator chicanes are tuned to a delay equivalent to the optical laser wavelength^29^.

After the undulator an X-band RF transverse deflector (TD) is used to streak the beam for time-resolved diagnostic purposes. The design of the structure allows for an arbitrary rotation of the streaking plane. Analysis of the streaked beam image yields information on the temporal properties of the electron beam as well as the FEL power profile with a resolution below 1 fs^36^.

The FEL light is transported to one out of two currently available soft X-ray endstations, which can operate alternatively without interfering with each other. The Maloja endstation has been in user operation since the beginning of 2022, the Furka endstation is under commissioning, and a third endstation, Diavolezza, is in the design phase. The beamlines can operate in pink or monochromatic mode, the latter realised with a variable-line-spacing spherical-grating monochromator. More information on the beamline optics can be found in ref. ^54^. Individual Kirkpatrick-Baez mirror systems downstream of the exit slits provide achromatic focusing of the beam to the experimental stations.

The Maloja experimental station is dedicated to atomic, molecular and non-linear X-ray physics and chemical dynamics. Maloja has a flexible configuration to enable innovative research on a wide range of targets from gas-phase atoms, molecules and clusters to liquids and nanoparticles. The broad suite of experimental techniques, including electron, ion and absorption spectroscopy as well as X-ray scattering allows users to trace electronic structure changes and nuclear rearrangement on their natural time scales. We achieve atomic spatial and femto- to attosecond temporal resolution by combining the state selectivity of X-rays with ultrashort pulse durations.

The Furka experimental station is dedicated to the study of quantum materials using time-resolved resonant inelastic and elastic X-ray scattering as well as X-ray absorption spectroscopy. In particular time-resolved resonant inelastic X-ray scattering will open scientific opportunities thanks to its capability to probe energy and momentum-resolved dynamics of elementary excitations involving different degrees of freedom in solids.

More detailed information on the Athos project can be found in refs. ^55,56^.

### Setup

The SwissFEL injector produces two duplicate electron bunches, separated by 28 ns, each of them with a typical charge of 200 pC. Both electron bunches are compressed in two bunch compression stages, called BC1 and BC2, and accelerated in linac 1 and 2 to an energy of 3.15 GeV. After linac 2, one of the two electron bunches continues straight to linac 3 and the Aramis undulator, while the other bunch is sent to the Athos beamline. The separation of the two bunches is achieved with a fast kicker, allowing parallel operation of Aramis and Athos at 100 Hz repetition rate. More information on the two-bunch operation at SwissFEL can be found in ref. ^57^.

The setup for the Athos bunch in the injector, linac 1, and linac 2, including compression in BC1 and BC2, is similar to the Aramis bunch, as described in^11^. In particular the emittance optimisation at the injector and after each bunch compression stage is equivalent for both bunches. Emittance values for the Athos bunch are comparable with those of the Aramis bunch^58^.

The RF settings of the Athos bunch can be tuned independently over a limited range thanks to a small step in phase and amplitude at the end of the RF pulse^57^. In the injector and linac 1 RF structures, the phase for the Athos bunch can be changed within ±1° with respect to the Aramis bunch, while keeping the same energy for both bunches. The exception is the X-band lineariser in the injector—here the phase can be varied independently by ±10°. We typically measure equivalent beam properties after BC1 for both bunches. By tuning the phase in linac 1 for bunch two only we can vary the compression in BC2 by about ±20 fs (rms values) with respect to the nominal pulse duration of around 25 fs (rms). The switchyard is designed to provide a certain degree of longitudinal dispersion to tune the pulse duration of the Athos bunch with its residual energy chirp. The current configuration provides a slight compression of the Athos bunch in the switchyard. This enables lower compression in BC2, thereby significantly reducing the impact of coherent synchrotron radiation in BC2 as well as in the switchyard. In addition to the linac 1 phase, the phase variation in the injector RF stations and the two EEHG chicanes give additional flexibility to tune the Athos bunch length.

There are seven one-metre corrugated structures before the Athos undulator capable of inducing energy and transverse chirps to the electron beam via wakefield excitation. These structures are meant to remove the residual energy chirp, required to compress the beam in BC2, of the Athos bunch (in Aramis, such a chirp is removed by the wakefields of linac 3). The dechirping functionality has been verified and presented in ref. ^57^. However, in standard operation the dechirper units are currently not employed. This is because the wakefields along the Athos beamline are stronger than anticipated and sufficient to remove the energy chirp of the Athos bunch.

Streaking the beam is fundamental for many operation modes, as shown before, e.g., for shortening the pulse in a tunable manner, for two-colour production in fresh-slice mode, or to produce high-power short pulses via the multi-stage fresh-slice amplification scheme. Streaking can be done with the transverse wakefields of the corrugated structures or by introducing dispersion with a quadrupole magnet in the switchyard. We typically use the second method since it results in lower beam losses. As a result of the tilt, the centre part of the beam stays on axis in the undulator and therefore produces FEL radiation, while the head and the tail parts of the bunch are off axis and do not lase. For the fresh-slice schemes, an additional correction is applied with dipole magnets before the undulator to shift the lasing fraction to the tail of the bunch. In the two-colour mode, dipole corrector magnets at the location of the two-colour chicane are tuned to align the head of the bunch in the second undulator half. For the multi-stage amplification scheme, as mentioned earlier, the transverse overlap between the FEL pulse and the fresh electrons is achieved with a dipole corrector magnet and the chicane offset at each intra-undulator section between two amplification stages (and the longitudinal overlap is assured by delaying the electron beam with the chicanes). In normal operation, aiming for maximum pulse energy, normal and skew quadrupole magnets in the switchyard are employed to correct any possible transverse beam tilts, following the work presented in ref. ^59^.

The electron beam trajectory is aligned with respect to the different components between the transfer line and the Athos undulator to minimise dispersion and wakefield effects. In the Athos undulator, procedures equivalent to the ones in Aramis, are implemented. Most importantly, a beam-based alignment (BBA) is carried out in the Athos undulator to ensure good transverse overlap between the electron and photon beams^60^. Furthermore, the alignment of each undulator module onto the electron trajectory in the four transverse degrees of freedom (horizontal and vertical offsets, pitch and yaw) is achieved by characterisation of spontaneous radiation filtered by a monochromator^61^. Due to the lack of a monochromator during commissioning, we instead use readily accessible X-ray absorption edges for this purpose. Finally, we taper the undulator modules after FEL saturation to maximise the final pulse energy. So far, we found that a second-order taper results in the highest output pulse energy. Applying a taper increases the final pulse energy by up to a factor of 4.

### Diagnostics

#### Electron diagnostics

Various electron beam diagnostics help to prepare the electrons for lasing and to keep stable performance with feedback loops. As in Aramis, the transverse emittances are reconstructed with the quadrupole scan technique as detailed in refs. ^62,63^. Besides the emittance measurements in the common part, which can be done for the Aramis and the Athos bunch, the emittance of the Athos bunch can be reconstructed before and after the Athos undulator. The transverse beam sizes, required for the emittance characterisation, are measured with screen monitors based on scintillating cerium-doped yttrium aluminium garnet (Ce:YAG) crystals^49^. The beam size determination at dispersive locations (in the switchyard and the Athos beam dump) is used to reconstruct the electron beam energy spread. An X-band RF TD structure with arbitrary rotation of the streaking plane and sub-femtosecond resolution^36^, installed after the undulator, is employed for time-resolved diagnostics of the electron beam. In particular, the LPS of the electrons can be measured with a screen in front of the beam dump. At this location the beam is both streaked in the horizontal direction and dispersed in the vertical plane. A corrugated structure, installed upstream of the RF TD station, can be used as an alternative temporal diagnostics of the electron beam^64^. The BBA is performed with cavity beam-position monitors (BPMs), which are able to measure the beam trajectory with sub-micrometre resolution. Beam-loss monitors ensure radiation safety during operation.

Beam-based feedback systems keep charge, energy, trajectory, compression, and arrival time of the Athos bunch constant, independently of the Aramis bunch. These systems therefore require diagnostics capable of distinguishing information from the two bunches. The charge of the electron beam is measured with integrating current transformers and the monopole cavities of the BPMs. Energy and trajectory feedback loops rely on BPM trajectory measurements at dispersive and non-dispersive locations, respectively. Similar feedback instances stabilising the compression are based on monitors installed after BC1 and BC2, which detect coherent edge radiation and coherent diffraction radiation, respectively. Energy and compression feedback actuators affect RF settings for the Athos bunch only.

#### Laser-based seeding diagnostics

Laser-based seeding diagnostics use screens and Al-coated Si crystals for spatial and temporal measurements. Screens are installed upstream and downstream of the U200 modulators for transverse overlap between the seed laser pulses and the electron beam. We employ a Cromox (chromium doped alumina Al_2_O_3_) or a Ce:YAG screen, depending on the seed laser wavelength. An Al-coated Si crystal acts as a mirror to deflect the optical laser light and produces optical transition radiation from the electron beam, both directed to a photodiode. The photodiode has a resolution of 15 ps and is used to establish a coarse longitudinal overlap between the electrons and the seed laser pulses. The fine tuning is done by scanning the seed laser delay stage while looking for a decrease of the FEL pulse energy.

#### Photon diagnostics

A range of photon diagnostics have been installed along the optical beamline from after the undulators to the endstation mirrors. They allow for single-shot measurements of all key X-ray pulse properties for optimisation of the FEL setup.

The diagnostics measuring the pulse intensity include silicon PIN diodes, ionisation gas cells and a gas detector for calibrated pulse energy measurements^37^. Spontaneous radiation detectors used for undulator calibration and alignment are located both in front of and behind the beamline monochromator. The X-ray beam spatial mode is monitored by a series of invasive scintillation screens downstream of all beamline optics and the photon beam trajectory is confirmed by a series of beamline apertures.

Single-shot X-ray spectral data are obtained from the beamline monochromator. The X-ray beam is spectrally dispersed with one of two available variable-line-spacing spherical gratings with 50 or 150 lines per millimetre mean line density. A 2D CMOS camera records images of the spectrally resolved X-ray beam from a screen inserted in the dispersive plane of the monochromator. In this way single-shot measurements of the spectral intensity profile are achieved with resolution better than 100 meV at 500 eV within a 2% bandwidth measurement window.

#### FEL power profile reconstruction

The FEL power profile is reconstructed by comparing the LPS of the electron beam for lasing-off and lasing-on conditions^65^. More specifically, we obtain the power profile by considering the slice energy spread increase when lasing is enabled^64^. For the power calibration we rely on the absolute pulse energy measured with the gas detector. Absolute time calibration is provided by the X-band TD. For the cases where the beam is tilted before the X-band TD, e.g., to generate short FEL pulses, the X-band TD adds up to the incoming tilt so that the final streaking becomes larger. For these configurations, the calibration is done assuming that the electron pulse duration does not change with respect to the standard case without initial beam streaking. We estimate time calibration errors to be 10% or less.

### Supplementary information

Peer Review File

## Data Availability

Relevant data supporting the key findings of this work are available within the article. All data generated during the presented work are available from the corresponding author upon request.
